# Kog1 Represses Lipid Accumulation in *Mucor circinelloides*: A Transcriptomic Analysis Across Nitrogen Conditions

**DOI:** 10.3390/jof12040266

**Published:** 2026-04-07

**Authors:** Zhen Wang, Ying Gao, Wenrui Dang, Lanlan Zhu, Huaiyuan Zhang

**Affiliations:** 1 School of Public Health, Qilu Medical University, Zibo 255300, China; 2 Baoding Institute for Food and Drug Control, Baoding 071030, China; 3 Colin Ratledge Center for Microbial Lipids, School of Agriculture Engineering and Food Science, Shandong University of Technology, 266 Xincun West Road, Zibo 255000, China

**Keywords:** oleaginous, *Mucor circinelloides*, transcriptome analysis, Kog1

## Abstract

Oleaginous microorganisms usually accumulate large amounts of lipids under nitrogen limitation and in a carbon-abundant environment. However, how cells sense changes in nitrogen and carbon levels in the culture medium remains a research hotspot. Previous studies have found that the target of rapamycin complex 1 (TORC1) plays a core role in lipid accumulation in oleaginous microorganisms. The results of the Kog1 (the member proteins of TORC1) knockout strain constructed earlier by our group showed that the Kog1 negatively regulated lipid accumulation in the oleaginous fungus *Mucor circinelloides*. In this study, transcriptomic analysis of the knockout and control strains under nitrogen-limited and nitrogen-sufficient culture was carried out to investigate significant differences in lipid accumulation. Kog1 knockout led to a significant decrease in cell dry weight and an increase in lipid content in *M. circinelloides*. The transcriptomic results showed that genes encoding the glyoxylic acid cycle and genes encoding acetyl-CoA carboxylase (ACC), fatty acid synthase (FAS), and Δ9 desaturase in lipid synthesis were upregulated to varying degrees under both conditions, indicating enhanced lipid metabolism that ultimately led to increased lipid accumulation. The knockout of the Kog1 gene also activated the pyruvate–acetaldehyde–acetate metabolic axis and significantly modified the branched-chain amino acid metabolic network, suggesting that Kog1 knockout reprograms the pathway of branched-chain amino acid synthesis and degradation, shifting the carbon flux from amino acid metabolism to acetyl-CoA accumulation. In addition, the gene encoding the SSK1p transcription factor, which participates in the nutrient stress response, was upregulated 41.9- and 51.9-fold in the Kog1 knockout strain compared with the control strain under nitrogen-limited and nitrogen-sufficient conditions, respectively.

## 1. Introduction

With the ongoing growth in global energy demand and the mounting environmental pressure from traditional fossil fuels, the development of renewable and clean alternative energy sources has become an important research direction in life science and biotechnology [1]. Microbial oils, which are triacylglycerols (TAGs) accumulated intracellularly by oleaginous microorganisms under specific conditions, are regarded as highly promising third-generation biofuel feedstocks due to their convertibility into biodiesel, the wide availability of raw materials, a short production cycle, and independence from arable land resources [2]. Various oleaginous microorganisms, such as yeasts, molds, and microalgae, can convert excess carbon sources into lipids under stress conditions such as nitrogen limitation and high carbon, with accumulation levels reaching over 20% of the cell dry weight, demonstrating great industrial application prospects [3]. However, the economic feasibility of microbial oil production remains limited by lipid yield, conversion efficiency, and cultivation cost [4]. Therefore, in-depth analysis of the molecular mechanism of lipid accumulation and identification of key regulatory factors have become core scientific issues for improving oil production performance.

Oleaginous microorganisms accumulate large amounts of lipids only under specific culture conditions, and the most common and effective method is nitrogen limitation in the culture medium [5]. According to the biochemical process of lipid synthesis, it can be divided into two stages in terms of time: the biomass growth stage and the lipid accumulation stage. During the lipid production process of oleaginous microorganisms, biomass growth and lipid accumulation are not synchronized over the fermentation time. The time lag leads to an overly long fermentation cycle in oleaginous microorganisms and to the inability to produce continuously. Moreover, the nitrogen-limited strategy adopted to achieve high lipid content reduces biomass, thereby affecting the final yield of microbial lipids.

To break free from the constraints of lipid production by oleaginous microorganisms under low-nitrogen conditions in the culture medium and to shorten the fermentation cycle, the most feasible approach is to simulate the signal transduction of nitrogen depletion within cells. However, the signal transduction mechanism after nitrogen depletion within oleaginous microorganisms remains unclear. Exploring how the signal of nitrogen depletion is conducted into the cell nucleus and regulates lipid accumulation is particularly important. Recent studies have found that the target of rapamycin complex 1 (TORC1) is of great significance in sensing extracellular nutritional status and can regulate downstream lipid metabolism processes [6]. The addition of rapamycin, a specific inhibitor of TORC1, during the culture of some oleaginous microorganisms can significantly increase lipid content in the cells. It was found that adding different concentrations of rapamycin during microbial cell culture can promote lipid synthesis in the cells, and some of these microorganisms are oleaginous. Adding rapamycin to *Saccharomyces cerevisiae* can promote lipid synthesis, but the promoting effect of lipid synthesis disappears after knocking out the *tor1* gene, indicating that rapamycin induces lipid accumulation through TORC1 [7]. After supplementing with leucine and adding 2 μg/mL rapamycin, the intracellular lipid content of the oleaginous yeast *Yarrowia lipolytica* Polf was more than twice that of the control group [8]. When the oleaginous yeast *Trichosporon oleaginosus* was cultured in the whole medium and 5 μM of rapamycin was added, the lipid content increased by 38% [6]. The addition of rapamycin, Torin1, and AZD8055 (rapamycin analogue with a stronger inhibitory effect on TOR) during the culture process of algae *Chlamydomonas reinhardtii* significantly increased the lipid content of the cells [9,10]. *S. cerevisiae* strains that were further knocked out of the gene *tor1* on the basis of *snf1* (AMP-dependent protein kinase, AMPK) significantly increased intracellular lipid content when cultured in a nitrogen-limited medium [11].

In addition to the core protein TOR, its regulatory protein Kog1 (Kontroller of growth 1, also known as Raptor (regulatory associated protein of TOR) in mammals) has also been found to have a high correlation between its expression level and fatty acid metabolism. It can also sense upstream nitrogen-containing signals for TOR and, at the same time, participate in regulating the energy state and stress response of cells under nitrogen-limiting conditions. Many animal and cell experiments have found that Raptor/Kog1 expression is highly related to fatty acid synthesis. In patients with rheumatoid arthritis, Raptor expression has been shown to be strongly correlated with alterations in lipid composition [12]. Consistently, murine models with Raptor knockout exhibit severe hypertriglyceridemia when challenged with a standard diet [13]. In addition, studies on the activity of TORC1 and fat accumulation in mice have found that the binding degree between raptor and TORC1 has a strong correlation with intracellular lipid synthesis [14]. Previous studies of this project team had shown that knockout of the Kog1 gene significantly increased intracellular lipid content (both under nitrogen-limitated and -sufficient conditions) [15].

To reveal the regulatory network of Kog1 in lipid metabolism of oleaginous microorganisms, this study constructed a Kog1 gene knockout strain and used the wild type as a control to conduct RNA-seq analysis. By comparing the whole-genome expression profiles of the two strains under oil production induction conditions, we focused on analyzing the expression changes of key genes in fatty acid synthesis, such as acetyl-CoA carboxylase (ACC), fatty acid synthase (FAS), diacylglycerol acyltransferase (DGA1), phospholipid:diacylglycerol acyltransferas (LRO1), β-oxidation, autophagy, and TORC1 downstream target genes, aiming to identify key functional pathways and candidate regulatory factors regulated by Kog1. Overall, this study provides new molecular evidence to understand the mechanism of the TORC1 signaling pathway in microbial oil production and offers a theoretical basis and gene targets for subsequent metabolic engineering to improve the oil yield.

## 2. Materials and Methods

### 2.1. Strains and Fermentation Conditions

The *M. circinelloides* Kog1 mutant strain Kog1^KO^-2 and the control strain MU1152, derived from *M. circinelloides* WJ11 (CCTCC No. M2014424), were constructed in the laboratory in the early stage [15]. The mutant strain Kog1^KO^-2 and the control strain MU1152 (1.0 × 10^7^ spores each) were separately placed in 500 mL baffled conical flasks containing 100 mL of K&R (Kendrick and Ratledge) nitrogen-limited and nitrogen-sufficient media [15]. They were cultured in the K&R medium in a shaking incubator at 28 °C and 130 rpm for 24 h, then transferred to a 1.5 L fermenter containing 1.0 L of modified K&R medium (glucose 80.0 g/L) for 120 h at a 10% (*v*/*v*) inoculation rate. The fermenter was maintained under the following conditions: 28 °C, 800 rpm, an air volume of 1.5 vvm, and the pH was adjusted to 6.0 using a 2.0 M NaOH solution. The components of the nitrogen-sufficient medium were the same as those of the modified K&R medium, but 20.0 g/L of diammonium tartrate and 15.0 g/L of yeast extract were additionally added, and 800 g/L of glucose was supplemented every 12, 24, 36, 48, and 72 h to ensure adequate glucose supply throughout the fermentation process.

### 2.2. Determination of Fermentation Parameters

Samples with different fermentation times were collected and filtered, the fresh mycelia were washed three times with distilled water and lyophilized, and the biomass of the samples was calculated using the differential weight method [16]. A total of 15 mg of the dried mycelium (reached a constant weight) was used to extract the total lipids using a chloroform/methanol (2:1, *v*/*v*) mixture with 15:0 stearic acid as the internal standard, according to a previously used method [15]. The extracted lipids were reacted with a 10% (*w*/*w*) HCl/methanol solution at 60 °C for 3 h to ensure methylation, then separated using hexane. Then, the lipid content and fatty acid composition were analyzed using a gas chromatograph (equipped with a DB-Waxetr chromatographic column, 30 m × 0.32 mm, 0.25 μm, Agilent 123-3232 model, Agilent, Santa Clara, CA, USA). The procedure was as follows: maintain at 80 °C for 10 min, then increase the temperature at a rate of 8 °C/min to 160 °C, then at a rate of 4 °C/min to 220 °C, and hold for 2 min. The lipid content of the *M. circinelloides* was calculated using the internal standard method. Each sample was analyzed three times in duplicate. The experiments were conducted with three biological replicates each, and the obtained data were presented as the mean (Mean) ± standard deviation (Standard Deviation) to ensure the reliability and reproducibility of the experimental results. An unpaired Student’s *t*-test was used to assess the statistical significance of differences between two groups. Differences with *p*-values lower than 0.05 were considered statistically significant.

### 2.3. Preparation of Transcriptomic Samples

To study the lipid accumulation characteristics of the *Kog1* gene knockout strain of *M. circinelloides* under different nitrogen source conditions, the fermentation broth collected after 24 h of cultivation (lipid accumulation occurs at the fastest rate) under nitrogen-limited (LNRap-K) and nitrogen-sufficient (HNRap-K) conditions was separately obtained. Meanwhile, the strain MU1152 was set as the control group (LN1152 and HN1152) under the same conditions. The sample processing procedure was as follows: 30 mL of the fermentation broth was placed in a pre-cooled glass beaker at 4 °C, filtered through a filter, and repeatedly washed with pre-cooled phosphate-buffered saline (PBS, pH 7.2–7.4) 3–5 times to remove residual medium. The washed mycelia were evenly divided into 2 mL centrifuge tubes, then immediately frozen in liquid nitrogen for 15–20 min for rapid freezing. Finally, they were transferred to a −80 °C refrigerator for storage and use in subsequent analyses, and total RNA was isolated using Trizol, according to the manufacturer’s instructions (Sangon Biotech, Shanghai, China).

### 2.4. Transcriptomic Analysis of Fermentation Products Between the Kog1 Knockout Strain and the Control Strain

For each fermentation sample obtained, a cDNA library was established. The low-quality, adapter-contaminated, and abnormally high base nitrogen content data obtained from the raw reads during sequencing were removed using SOAPnuke software (version 1.4.0) to ensure the reliability of the results [17]. The filtered data were then aligned to the genomic sequence using Bowtie2, and the expression levels of the mutant and control strain fermentation products were calculated using RSEM (version 1.2.12) [18]. Based on the negative binomial distribution principle, the differentially expressed genes (DEGs) between the fermentation product groups were identified using the DEseq2 method, and then DEG detection was carried out [19].

Based on the gene expression levels of the fermentation samples, through quantitative gene analysis, principal component analysis, correlation analysis, and differential gene screening methods, the significant sufficiency analysis of DEGs in the selected samples of the KEGG (Kyoto Encyclopedia of Genes and Genomes) pathway was conducted using the BioCloud platform of Zhongke Xin Life Bioinformatics (https://bio-cloud.aptbiotech.com). Combined with gene function annotation and official classification, the DEGs were classified into biological pathways, and sufficiency analysis was performed using the phyper function in R. Subsequently, the *p*-value was calculated using the false discovery rate (FDR) and corrected. Generally, pathways with a Q-value ≤ 0.05 are considered significant for sufficiency.

## 3. Results

### 3.1. The Cell Growth and Lipid Accumulation of Kog1 Mutant Strain Under Nitrogen-Limited and Nitrogen-Sufficient Conditions

**Figure 1:**
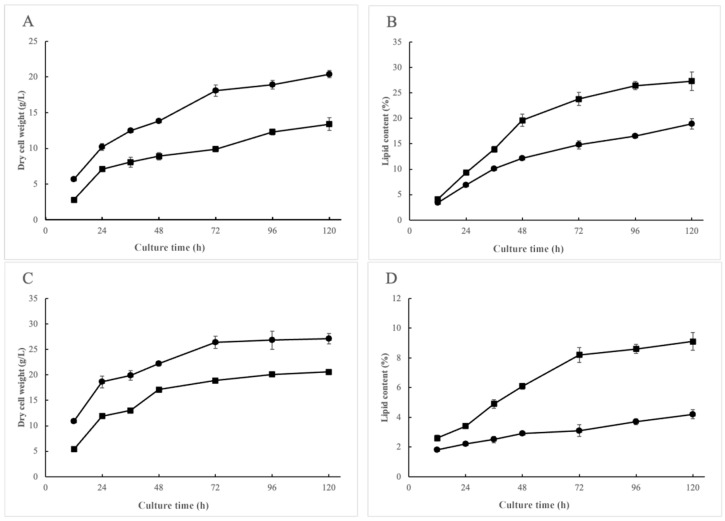
Comparison the dry cell weight and lipid content between Kog1^KO^-2 (square) and MU1152 (circle) under nitrogen-limited (**A**,**B**) and nitrogen-sufficient (**C**,**D**) conditions. The experiments were conducted with three biological replicates each, and the obtained data were presented as the mean ± SD. An unpaired Student’s *t*-test was used to assess the statistical significance of differences between two groups. Differences with *p*-values lower than 0.05 were considered statistically significant.

The Kog1 knockout strain and the control strain showed significant differences in biomass and lipid content when cultured in nitrogen-limited and nitrogen-sufficient culture, respectively. The Kog1 knockout significantly inhibited cell growth. From the early stage of fermentation, the dry cell weight of the Kog1 knockout strain was lower than that of the control group. Under nitrogen-limited conditions, the dry cell weight decreased from 20.4 to 13.4 g/L, a reduction of 34.3% (Figure 1A), while the knockout of Kog1 led to a significant increase in intracellular lipid content, which was 44.4% higher than that of the control strain, from 18.9% to 27.3% (Figure 1B), the overall lipid yield remained unaffected. In nitrogen-sufficient culture, the cell dry weight decreased from 27.1 to 20.6 g/L, decreased by 24.0% (Figure 1C), and the lipid content also increased significantly, from 4.2% to 9.1% (Figure 1D).

### 3.2. RNA Sequencing and Analysis of Kog1 Knockout Strains Under Nitrogen-Limited and Nitrogen-Sufficient Conditions

After constructing the RNA-seq libraries, sequencing was performed on three biological replicates of samples at each time point, followed by nucleotide analysis. The raw sequencing data (Raw Reads) were processed through a strict quality control process, resulting in high-quality Clean Reads. The quality control steps included (1) removing adapter sequences, (2) filtering reads containing more than 10% unknown bases (N), and (3) eliminating low-quality reads. It is noteworthy that the Clean Read yield of both mutant strains and wild-type control strains remained at a very high level under both nitrogen-sufficient and nitrogen-limited conditions, accounting for more than 99.5% of the original data, indicating excellent sequencing data quality and an effective processing procedure. Additionally, the Q30 quality score is a quality indicator for individual bases, representing an error rate of 1/1000 (each 1000 bp sequencing read may contain one error) as the quality control benchmark. Typically, most benchmark sequencing produces a Q30 score above 70–80% as an indicator of a successful sequencing run [20]. In this study, the average Q30 read range was 96.9–97.0%. These data indicate that the error rate of RNA-Seq sequencing data is low, the original sequence reads have high reliability and quality that meet statistical requirements, and they can be further analyzed in experiments.

### 3.3. Analysis of Gene Differential Expression in Kog1 Knockout Strains Under Nitrogen-Limited and Nitrogen-Sufficient Conditions

**Figure 2:**
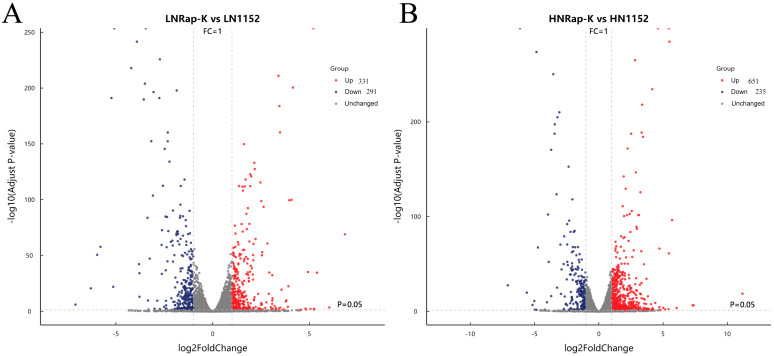
A scatter plot of Kog1^KO^-2 and MU1152 under nitrogen-limited (**A**) and nitrogen-sufficient conditions (**B**). LNRap-K, the Kog1 knockout strain under nitrogen-limited conditions; LN1152, MU1152 under nitrogen-limited conditions; HNRap-K, the Kog1 knockout strain under nitrogen-sufficient conditions; HN1152, the MU1152 under nitrogen-sufficient conditions; Up, upregulated genes; Down, downregulated genes. DEGs had log2FC > 0 and FDR < 0.05.

To study the gene regulatory mechanism of lipid accumulation in *M. circinelloides* Kog1 knockout under nitrogen-limited and -sufficient conditions at the molecular level, the original sequencing data for the samples were first purified and then compared with the reference genome. Based on RNA-seq analysis and with a threshold of adjusted *p* ≤ 0.05, DEGs were identified. With a threshold of 2-fold change (i.e., |log2 FoldChange| ≥ 1) and FDR < 0.05, the number of upregulated and downregulated DEGs was determined. After conducting differential expression analysis between Kog1^KO^-2 and the control strain MU1152, it was found that 622 DEGs significantly changed under nitrogen-limited conditions, among which 331 genes were upregulated and 291 genes were downregulated (Figure 2A); 886 DEGs significantly changed under nitrogen-sufficient conditions, among which 651 genes were upregulated and 235 genes were downregulated (Figure 2B).

### 3.4. KEGG Sufficiency Analysis of Kog1 Knockout Strains Under Nitrogen-Limited and Nitrogen-Sufficient Conditions

KEGG pathway annotation and sufficiency analysis were conducted on the DEGs to investigate the main changes in the biological pathways involved in the fungus *M. circinelloides* WJ11 after knockout of Kog1 under nitrogen-limited and nitrogen-sufficient conditions. Up to 11,360 expressed genes could be annotated, and 304 metabolic pathways were obtained. With a Q-value < 0.05 and *p* ≤ 0.05 as the threshold, compared with the control strain, a total of 22 metabolic pathways were significantly enriched under nitrogen limitation after knockout of Kog1, and 130 DEGs showed upregulation or downregulation; under nitrogen-sufficient conditions after knockout of Kog1, a total of 26 metabolic pathways were significantly enriched, and 142 DEGs showed upregulation or downregulation. The results of KEGG sufficiency analysis showed that under nitrogen-limited and nitrogen-sufficient conditions, metabolic pathways (accounting for 73.1% and 61.3%, respectively), microbial metabolism under various environments (accounting for 21.5% and 17.6%, respectively), and pyruvate metabolism (accounting for 8.5% and 6.3%, respectively) were significantly enriched. However, branched-chain amino acid biosynthesis (accounting for 3.1%) was only enriched under nitrogen limitation, and branched-chain amino acid degradation (accounting for 5.6%) was only significantly enriched under nitrogen-sufficient conditions.

### 3.5. The Influence of Kog1 Knockout Under Nitrogen-Limited and Nitrogen-Sufficient Conditions on the Expression of Genes Involved in the Lipid Synthesis Pathway

**Figure 3:**
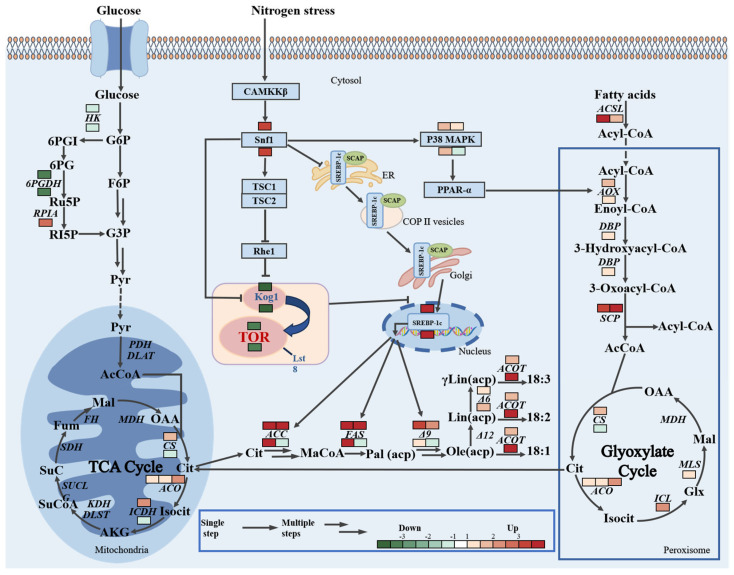
Transcriptomic features of metabolic pathways in Kog1 knockout strain Kog1^KO^-2 compared with the control strain MU1152 under nitrogen-limited and nitrogen-sufficient conditions. The changes shown above for enzymes and proteins are under nitrogen-limited conditions, while those shown below are under nitrogen-sufficient conditions. Genes without annotations did not show significant changes in expression. Down, downregulated gene; Up, upregulated gene. DEGs had log2FC > 0 and FDR < 0.05.

To study the role of Kog1 knockout in lipid metabolism, further analysis was conducted on the DEGs identified between the knockout strain Kog1^KO^-2 and the control strain MU1152 under nitrogen-limited and nitrogen-sufficient conditions (Figure 3). Under nitrogen-limited conditions, compared with the control strain, the gene expressions encoding key enzymes, hexokinase (HK) of the glycolysis pathway and 6-phosphogluconate dehydrogenase (6PGDH) of the pentose phosphate pathway were, respectively downregulated 1.4- and 3.3-fold, while the gene expressions encoding citrate synthase (CS) and isocitrate dehydrogenase (ICDH) from the tricarboxylic acid (TCA) cycle were, respectively, upregulated 1.9- and 2.1-fold. Under nitrogen-sufficient conditions, the gene expressions encoding HK and 6PGDH in the mutant were downregulated, and the trend was similar to that observed under nitrogen-limited conditions. However, unlike under nitrogen-limited conditions, the gene expressions encoding CS and ICDH showed a downregulation trend under nitrogen-sufficient conditions, with reductions of 2.5- and 2.3-fold, respectively. Additionally, the transcriptome data showed that after knockout of Kog1, under both nitrogen-limited and nitrogen-sufficient conditions, the genes encoding enzymes involved in lipid biosynthesis, such as ACC, FAS, fatty acid Δ9 desaturase (Δ9), fatty acid Δ6 desaturase (Δ6), and acetyl-CoA thioesterase (ACOT), were upregulated to varying degrees in both conditions; meanwhile, the genes encoding the fatty acid β-oxidation process involved in lipid catabolic metabolism, such as p38 mitogen-activated protein kinase (p38 MAPK) and acyl-CoA oxidase (AOX), also showed upregulation to varying degrees.

Metabolite annotation: G6P, Glucose-6-phosphate; F6P, Fructose-6-phosphate; G3P, Glycerol-3-phosphate; 6PGI, Gluconate-6-phosphate ester; 6PG, Gluconate-6-phosphate; Ru5P, Ribulose-5-phosphate; Ri5P, Ribose-5-phosphate; Pyr, pyruvic acid; Acetyl-CoA, Acetyl-CoA; Cit, citric acid; Isocit, isocitric acid; AKG, α-Ketoglutaric acid; SuCoA, Succinyl-CoA; SuC, succinic acid; Fum, fumaric acid; Mal, malic acid; OAA, oxaloacetic acid; MaCoA, Propionyl-CoA; Pal, palmitoyl; Ole(acp), Oleoyl (acyl carrier protein); Lin(acp), linolenyl (acyl carrier protein); γLin(acp), γ-Linolenoyl (acyl carrier protein); Enoyl-CoA, Enoyl-CoA; 3-Hydroxyacyl-CoA, 3-Hydroxyacyl-CoA; 3-Oxyl-Acyl-CoA, 3-Oxyl-Acyl-CoA; Glx, acetaldehyde acid.

Enzyme annotation: HK, hexokinase; 6PGDH, 6-Phosphogluconate dehydrogenase; RPLA, ribulose-5-phosphate isomerase, PDH, pyruvate dehydrogenase; DLAT, dihydroxylamine acetyltransferase; CS, citrate synthase; ACO, ursolic acid; ICDH, isocitrate dehydrogenase; KDH, α-Ketoglutaric acid dehydrogenase; DLST, dihydroxysuccinyl-lysine residue succinyltransferase; SUCLG, succinyl-CoA ligase; SDH, succinate dehydrogenase; FH, fumaric acid; MDH, malic dehydrogenase; ACC, Acetyl-CoA carboxylase; FAS, fatty acid synthase; Δ9, fatty acid Δ9 desaturase; Δ6, fatty acid Δ6 desaturase; ACOT, Acetyl-CoA thioesterase; ACSL, Acyl-CoA synthase; AOX, Acyl-CoA oxidase; DBP, D-Bifunctional protein; SCP, sterol carrier protein; ICL, isocitrate; MLS, malic acid synthase.

### 3.6. Various Sources of Acetyl-CoA for Fatty Acid Synthesis in Kog1 Knockout Strains Under Nitrogen-Limited and Nitrogen-Sufficient Conditions

**Figure 4:**
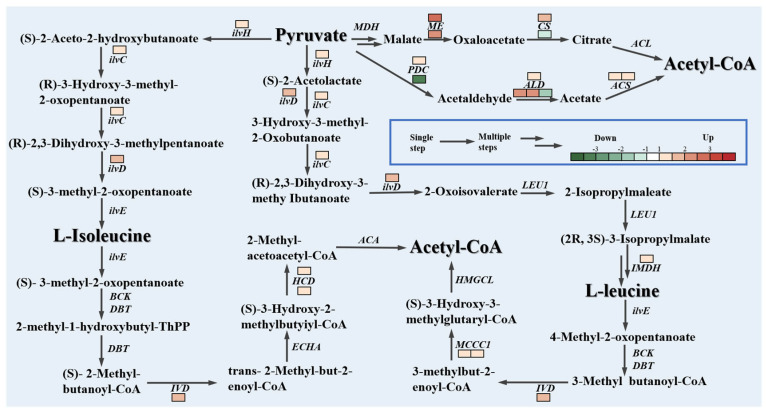
Transcriptomic features of the acetyl-CoA metabolic pathway in the *Kog1* knockout strain Kog1^KO^-2 compared with the control strain MU1152 under nitrogen-limited and nitrogen-sufficient conditions. Down, downregulated gene; Up, upregulated gene. DEGs with log2FC > 0 and FDR < 0.05.

Acetyl-CoA is the central precursor in fatty acid biosynthesis. The sufficiency of precursor substances significantly impacts lipid accumulation. Transcriptomic analysis indicates that under nitrogen-limited conditions, compared with the control strain MU1152, after the Kog1 gene was knocked out, the expression levels of genes encoding pyruvate decarboxylase (PDC), acetaldehyde dehydrogenase (ALD), and acetyl-CoA synthase (ACS) were significantly increased; the gene encoding ME was upregulated 2.9- and 2.5-fold under nitrogen-limited and nitrogen-sufficient conditions, respectively. These data suggest that cytoplasmic pyruvate is converted into the precursor acetyl-CoA through multiple reactions (Figure 4). Additionally, transcriptomic analysis revealed that Kog1 gene knockout promoted the synthesis and degradation metabolic pathways for leucine and isoleucine under nitrogen-limited conditions. The expression level of the gene encoding the key regulatory subunit of acetolactate synthase (ilvH) was upregulated 2.2-fold, while the expression of the gene responsible for encoding 3-hydroxyacyl-CoA dehydrogenase (HCD) was significantly increased 2.3-fold.

Under nitrogen-sufficient conditions, compared with the control strain MU1152, the gene encoding PDC was significantly downregulated after the Kog1 gene was knocked out. The expression levels of the three genes encoding ALD increased significantly by 2.4- and 5.3-fold, respectively, while the expression of the gene encoding the synthesis pathway of leucine and isoleucine decreased 3.8-fold. There were no significant changes in the genes encoding the synthesis pathways of leucine and isoleucine, but the genes encoding the key enzymes in the metabolic process of degrading leucine and isoleucine to generate acetyl coenzyme A were significantly upregulated. The expression level of the IVD gene encoding medium-chain-specific acyl-CoA dehydrogenase was significantly upregulated by 3.7-fold. The two genes encoding the α subunits of 3-methylcrotonyl-CoA carboxylase (MCCC1) were significantly upregulated by 2.0- and 2.1-fold, respectively, and the gene encoding HCD was significantly upregulated (2.5-fold) under nitrogen-sufficient conditions, which was slightly higher than that under nitrogen-limiting conditions (2.3-fold).

Enzyme annotation: ilvH, glyoxal dehydratase; ilvC, glyoxal reductoisomerase; ilvD, dihydroxy acid dehydratase; ilvE, branched-chain amino transferase; BCK, branched-chain α-keto acid dehydrogenase complex; DBT, dihydrostearoyl acetyltransferase; IVD, isopentenyl coenzyme A dehydrogenase; ECHA, Enol coenzyme A hydratase; HCD, hydroxyacyl coenzyme A dehydrogenase; ACA, acetylacetyl coenzyme A lyase; HMGCL, 3-hydroxy-3-methylglutaryl coenzyme A lyase; MCCC1, 3-methylglutaryl coenzyme A carboxylase 1; ME, malic enzyme; IMDH, isocitrate dehydrogenase; LEU1, leucine synthase 1; PDC, pyruvate dehydrogenase complex; ALD, acetaldehyde dehydrogenase; ACS, acetyl coenzyme A synthase; ACL, ATP-lycoglycylase; CS, citrate synthase; PC, pyruvate carboxylase.

### 3.7. Transcriptional Analysis of Differentially Expressed Genes in Kog1 Knockout Strains Under Nitrogen-Limited Conditions

After a comprehensive review of transcriptome data under nitrogen-limited conditions, DEGs with mRNA levels upregulated or downregulated by 15-fold at 24 h after Kog1 gene knockout were selected for simplified analysis (Table 1). The top 16 upregulated DEGs included the protease 4 (aspartic protease) of the Rhizopus genus involved in protein processing (ID: HMPREF1544_11888), which was upregulated 114.1-fold. Two genes encoding transcription factors of the two-component regulatory agent SSK1p (ID: HMPREF1544_01989, HMPREF1544_07917) were upregulated 65.2- and 41.9-fold, respectively. The transmembrane amino acid transporter domain protein encoding genes involved in the fungal signal transduction process and playing a key role in stress responses (ID: HMPREF1544_10544), glycosylphosphatidylinositol (GPI) (ID: HMPREF1544_11645), and CCHC-type zinc finger transcription factor (ID: HMPREF1544_12387) were upregulated 33.3-, 23.1-, and 15.5-fold, respectively. The gene encoding the sphingosine transferase involved in lipid metabolism (lag1, ID: HMPREF1544_09775) was upregulated 30.3-fold; the gene encoding the sterol carrier protein (SCP) (ID: HMPREF1544_12290) was upregulated 27.4-fold. In addition, the genes encoding the common subunits of RNA polymerase I, II, and III, which are involved in assembly, stability, and catalytic activity of the polymerase (ID: HMPREF1544_10206), were upregulated by 15.1-fold, thereby affecting the synthesis of rRNA, mRNA, and tRNA.

The first seven downregulated DEGs include three genes related to the strain’s growth that were significantly downregulated. Specifically, the gene encoding P-type ATPase for proton extrusion in the plasma membrane (Pma1) (ID: HMPREF1544_02897) was significantly downregulated 37.5-fold; the gene encoding the OPT family small oligopeptide transporter (ID: HMPREF1544_03166) was downregulated 33.9-fold. The genes encoding the 3-keto steroid reductase (ID: HMPREF1544_06346), the gene encoding the C5HC2 type zinc finger transcription factor (ID: HMPREF1544_11142), and the gene encoding the gig inhibitor (ID: HMPREF1544_02900) were downregulated 78.5-, 62.6-, and 18.5-fold, respectively.

### 3.8. Transcriptional Analysis of Differentially Expressed Genes in Kog1 Knockout Strains Under Nitrogen-Sufficient Conditions

When the Kog1 gene was knocked out under nitrogen-sufficient conditions, the number of DEGs whose mRNA levels were significantly upregulated or downregulated by at least 15-fold was higher than under nitrogen-limiting conditions (Table 2). Specifically, among the top 16 significantly upregulated genes under nitrogen-sufficient conditions, there was a gene encoding the peptidase M20 domain protein 2 (ID: HMPREF1544_07585), with an upregulation amplitude of 157.8 fold; a gene encoding the motor protein-like protein involved in vesicle transport and cell morphology maintenance (ID: HMPREF1544_07875), with 43.6-fold upregulation; and a gene encoding acyl-CoA thioesterase (Acyl-CoA thioesterase, ACOT) related to lipid accumulation (ID: HMPREF1544_05389), with a significant upregulation of 42.9-fold. This might have prompted the mutant to increase its lipid accumulation. In addition, transcriptomic analysis also revealed that the gene encoding the S-adenosyl-L-methionine-dependent methyltransferase (S-adenosyl-L-methionine-dependent methyltransferase, SAM) involved in regulating gene expression and protein modification (ID: HMPREF1544_05389) was upregulated 32.2-fold; the gene encoding the COP9 signalosome (COP9 signalosome, CSN) subunit involved in integrating different signaling pathways (ID: HMPREF1544_08161) was upregulated 26.4-fold; and the gene encoding the amino acid permease/SLC12A domain protein (ID: HMPREF1544_02399) was upregulated 26.3-fold. Moreover, the gene encoding the lactulose phosphate ribose transferase (ID: HMPREF1544_02749) and the transcription factor encoding the two-component response regulator SSK1p (ID: HMPREF1544_07917) showed more significant upregulation under nitrogen-sufficient conditions compared to under nitrogen-limiting conditions, with upregulation of 43.5- and 51.9-fold, respectively.

The eight downregulated differentially expressed genes include the gene encoding 3-keto steroid reductase (ID: HMPREF1544_06346), the gene encoding C5HC2 type zinc finger transcription factor (ID: HMPREF1544_11142), and the gene encoding gig inhibitor (ID: HMPREF1544_02900). These genes were downregulated 48.5-fold, 35.7-fold, and 28.8-fold, respectively. Additionally, among these differentially expressed genes with significant changes in transcription levels, some genes encode hypothetical proteins. Among them, the hypothetical protein (ID: HMPREF1544_07916) was upregulated in both conditions, while the hypothetical protein (ID: HMPREF1544_10105) was downregulated in both conditions. These findings provide important clues for subsequent studies on the growth, development, and metabolic changes of *M. circinelloides*.

## 4. Discussion

The appropriate regulation of gene transcription is crucial for cells to grow under nutrient-sufficient conditions and survive under nutrient-limited conditions [21]. In a previous study, we found that after knocking out Kog1, compared with the control strain MU1152, the lipid content of the mutant strain increased to varying degrees under nitrogen-limited and nitrogen-sufficient conditions in the oleaginous fungus *M. circinelloides* [15]. To clarify the molecular mechanism of the specific metabolic process of the lipid biosynthesis pathway after knocking out Kog1, the transcriptome was analyzed to determine the lipid accumulation and metabolic pathways of the mutant strain *M. circinelloides* Kog1^KO^-2 and the control strain MU1152 under nitrogen-limited and nitrogen-sufficient conditions. The expression of the gene encoding 6PGDH in the pentose phosphate pathway was downregulated under nitrogen-limited and nitrogen-sufficient conditions (Figure 3). This is contrary to the results previously reported in *Rhodosporidium toruloides* and *Y. lipolyitica*, where the expression levels of the gene encoding 6PGDH in the lipid accumulation process were significantly increased [22,23]. However, we observed that the gene encoding ME was upregulated under nitrogen-limited and nitrogen-sufficient conditions (Figure 4). Previous studies have shown that ME and 6PGDH in the pentose phosphate pathway are important sources of NADPH for fatty acid biosynthesis [24,25,26]. This suggests that after knocking out Kog1, ME may play a primary role in providing NADPH to promote lipid accumulation. In addition, the gene encoding HK was downregulated in the mutant strain under nitrogen-limited and nitrogen-sufficient conditions, while the changes in the genes related to the TCA cycle pathway were not consistent under the two conditions (Figure 3). The downregulation of glycolysis pathway genes is conducive to cells preserving energy and redirecting carbon flux [27], and the TCA cycle provides essential precursors for nitrogen metabolism [23,28], which is consistent with the RT-qPCR results published in a previous study [15]. These results suggested that the knockout of the Kog1 gene was associated with the potential reprogramming of the carbon flux and nitrogen cycling system within the cells. Lipid production in oleaginous microorganisms involves the dynamic interaction of the fatty acid biosynthesis pathway and the fatty acid degradation pathway [29]. Transcriptome data revealed that the genes encoding p38 MAPK and fatty acid oxidation-related AOX were upregulated to varying degrees under nitrogen-limited and nitrogen-sufficient conditions (Figure 3). The knockout of Kog1 activated Snf1 and stimulated the transcriptional activity of the key regulatory factor for fatty acid metabolism—peroxisome proliferator-activated receptor (PPARα)—through an evolutionarily conserved p38 MAPK, inducing the expression of PPARα target genes AOX [30,31,32], promoting the β-oxidation of fatty acids in the lipid breakdown metabolic process, and further upregulating the genes encoding the intermediates of the acetoacetyl-CoA cycle. Since the supplementary metabolic pathway (acetoacetyl-CoA cycle) can generate intermediate products of the TCA cycle (citrate), it drives the redistribution of central carbon flux to the fatty acid synthesis pathway [29,33], resulting in the upregulation of the genes encoding key enzymes of lipid synthesis ACC, FAS, and Δ9 under both culture conditions (Figure 3), which is consistent with the previous RT-qPCR results. Moreover, in the DEGs under nitrogen-limited conditions, the gene *lag1* encoding the sphingosine N-acyltransferase involved in the fatty acid biosynthesis process was significantly upregulated [34], and the gene *scp* encoding the β-oxidation of hydrogen peroxidase in the β-oxidation process in the peroxisome [35] was also significantly upregulated (Table 2). These data indicate that Kog1 knockout may lead to interactions between lipid synthesis and metabolism during the growth and development of *M. circinelloides*. Acetyl-CoA is an important hub in microbial bioenergetics and synthetic metabolism and is also the central precursor for fatty acid biosynthesis. The supply of this key metabolic intermediate is crucial for fatty acid biosynthesis [36]. Previous studies have shown that the decarboxylation process of pyruvate and the synthesis and degradation of branched-chain amino acids (BCAAs) can provide the precursor Acetyl-CoA [36,37]. The gene encoding ALD in the mutant was significantly upregulated under nitrogen-limited and nitrogen-sufficient conditions, thereby increasing the precursor acetyl-CoA provided by pyruvate–acetaldehyde–acetic acid for lipid accumulation in the cytoplasm [38] (Figure 4). Additionally, the gene encoding ilvH in the branched-chain amino acid synthesis pathway was significantly upregulated only under nitrogen-limited conditions. Previous studies have shown that overexpression of ilvH can lead to significant accumulation of acetyl-CoA [39]. The gene encoding MCCC1 in the branched-chain amino acid degradation pathway was significantly upregulated only under nitrogen-sufficient conditions, thereby allowing Acetyl-CoA to accumulate [40]. This indicates that the knockout of Kog1 was correlated with the reprogramming of branched-chain amino acid synthesis and degradation metabolism to provide survival advantages for the mutant, while redistributing carbon flux from the amino acid metabolism pathway to the accumulation of acetyl-CoA, and introducing different metabolic pathways in response to nutritional stress or amino acid excess to promote fatty acid biosynthesis. Furthermore, we noticed that the gene encoding SSK1p was significantly upregulated under both nitrogen-limited and nitrogen-sufficient conditions. Previous studies have shown that SSK1p is involved in regulating the response of fungi to stress conditions such as nutrient stress [41]. It is speculated that the knockout of Kog1 makes the cell more sensitive to changes in the external nutritional environment, enabling it to respond to external nutrient signals and thereby survive for a long time under stress conditions.

Transcriptomic analysis was conducted to assess lipid accumulation, gene changes, and functional annotations in the metabolic pathways of the mutant strain *M. circinelloides* Kog1^KO^-2 and the control strain MU1152 after 24 h of cultivation under nitrogen-limited and nitrogen-sufficient conditions. KEGG sufficiency analysis revealed that the mutant strain exhibited unique mechanisms of nutritional perception and response. It regulated the expression level of the key enzyme ME, which is responsible for NADPH generation, to promote lipid accumulation. It also dynamically regulated the carbon-nitrogen metabolic balance, profoundly influencing the interactions between lipid synthesis and decomposition metabolic networks during the growth and development of *M. circinelloides*. Notably, the knockout of the Kog1 gene significantly altered the branched-chain amino acid metabolic network: under nitrogen limitation, genes in the branched-chain amino acid biosynthesis pathways were significantly enriched, whereas under nitrogen sufficiency, genes in the degradation pathways were significantly upregulated. Further analysis revealed that genes involved in the pyruvate–acetaldehyde–acetic acid pathway were upregulated in the mutant, suggesting a potential activation of this axis and a possible reallocation of carbon flux to support fatty acid biosynthesis under stress conditions, which may contribute to adaptive metabolic homeostasis. Correlation analysis between metabolomics and transcriptomics can further improve understanding of the molecular mechanisms regulating lipid metabolism in oleaginous microorganisms.

## Figures and Tables

**Table 1:** Log_2_ fold change (FC) of DEGs 24 h after deletion of Kog1 under nitrogen-limited conditions.

	Gene ID	Description	FPKM Value		Log_2_FC
			MU1152	Kog1^KO^-2	
Up	HMPREF1544_11888	Rhizopuspepsin-4	0.08	10.43	6.83
	HMPREF1544_01989	Two-component response regulator SSK1p	0.00	0.69	6.03
	HMPREF1544_07917	Two-component response regulator SSK1p	0.16	7.63	5.39
	HMPREF1544_02926	Hypothetical protein PHYBLDRAFT_71060	0.00	1.54	5.24
	HMPREF1544_05215	Hypothetical protein HMPREF1544_05215	0.00	0.21	5.23
	HMPREF1544_02749	Orotate phosphoribosyltransferase	78.36	3204.03	5.21
	HMPREF1544_10544	Transmembrane amino acid transporter protein-domain-containing protein	0.00	0.08	5.06
	HMPREF1544_09775	Sphingosine N-acyltransferase Lag1	0.13	4.26	4.92
	HMPREF1544_01909	Orotate phosphoribosyltransferase	0.00	0.21	4.83
	HMPREF1544_12290	Sterol carrier protein 2	0.01	0.25	4.79
	HMPREF1544_11042	Hypothetical protein HMPREF1544_11042	0.00	0.07	4.56
	HMPREF1544_05286	Golgi transport complex subunit 3 (GTC3)	0.00	0.19	4.56
	HMPREF1544_11645	Glycosylphosphatidylinositol anchor biosynthesis	0.00	0.16	4.53
	HMPREF1544_07916	-	1.03	19.18	4.08
	HMPREF1544_12387	CCHC-type zinc finger transcription factor	3.95	68.14	3.96
	HMPREF1544_10206	Subunit common to RNA polymerases I, II, and III	0.64	10.48	3.91
Down	HMPREF1544_03348	3-keto-steroid reductase	5.39	0.00	−7.09
	HMPREF1544_06346	3-keto-steroid reductase	2.21	0.03	−6.29
	HMPREF1544_10105	C5HC2-type zinc finger transcription factor	118.26	2.09	−5.97
	HMPREF1544_11142	Plasma-membrane proton-efflux P-type ATPase	3.09	0.06	−5.80
	HMPREF1544_02897	OPT family small oligopeptide transporter	14.61	0.43	−5.23
	HMPREF1544_03166	Gig suppressor	90.32	2.95	−5.08
	HMPREF1544_02900	3-keto-steroid reductase	54.32	3.25	−4.21

Note: Down, downregulated gene; Up, upregulated gene. log2FC was calculated using Deseq2 software 3.20.

**Table 2:** Log_2_ fold change (FC) of DEGs 24 h after deletion of Kog1 under nitrogen-sufficient conditions.

	Gene ID	Description	FPKM Value		Log_2_FC
			MU1152	Kog1^KO^-2	
Up	HMPREF1544_10224	-	0.00	0.50	7.36
	HMPREF1544_07585	Peptidase M20 domain-containing protein 2	0.00	3.27	7.30
	HMPREF1544_07917	Two-component response regulator SSK1p	0.41	22.49	5.70
	HMPREF1544_07916	-	1.39	66.16	5.49
	HMPREF1544_07875	Kinesin-like protein	0.25	11.62	5.45
	HMPREF1544_02749	Orotate phosphoribosyltransferase	33.02	1507.77	5.44
	HMPREF1544_09896	Acyl-CoA thioesterase	0.00	0.10	5.42
	HMPREF1544_05389	S-adenosyl-L-methionine-dependent methyltransferase	0.01	0.43	5.01
	HMPREF1544_08161	COP9 signalosome (CSN) subunit	0.00	0.20	4.72
	HMPREF1544_02399	Amino acid permease/SLC12A domain-containing protein	0.17	4.81	4.72
	HMPREF1544_09941	Helix–loop–helix DNA-binding domain-containing transcription factor	0.17	4.56	4.71
	HMPREF1544_07874	CUE domain-containing protein	0.06	1.31	4.39
	HMPREF1544_00413	-	0.02	0.32	4.21
	HMPREF1544_04208	Helix–loop–helix DNA-binding domain-containing transcription factor	0.01	0.18	4.19
	HMPREF1544_10674	ATP-binding cassette transporter snq2	0.07	1.35	4.18
	HMPREF1544_08626	Cyclin	0.03	0.59	4.17
Down	HMPREF1544_10105	-	116.99	1.77	−6.12
	HMPREF1544_06346	3-keto-steroid reductase	1.85	0.04	−5.60
	HMPREF1544_11142	C5HC2-type zinc finger transcription factor	0.34	0.01	−5.16
	HMPREF1544_02900	Gig suppressor	59.19	2.15	−4.85
	HMPREF1544_02041	Hemerythrin hhe cation binding subfamily protein	259.53	10.25	−4.73
	HMPREF1544_11747	Coth protein-domain-containing protein	0.11	0.01	−4.20
	HMPREF1544_12354	-	2.00	0.13	−4.03
	HMPREF1544_01972	F-box domain-containing protein	4.45	0.30	−3.95

Note: “-” indicates that genes encoding unknown proteins have no KEGG annotation or annotation description. Down, downregulated gene; Up, upregulated gene. log2FC was calculated using Deseq2 software.

## Data Availability

The original contributions presented in this study are included in the article. Further inquiries can be directed to the corresponding authors.
